# Bright, stable, and water-soluble CuInS_2_/ZnS nanocrystals passivated by cetyltrimethylammonium bromide

**DOI:** 10.1186/s11671-015-0836-0

**Published:** 2015-03-20

**Authors:** Jun Lee, Chang-Soo Han

**Affiliations:** School of Mechanical Engineering, Korea University, 145 Anam-ro, Seoul, Korea

**Keywords:** CuInS_2_/ZnS nanocrystals, CTAB, Photostability, Cold treatment

## Abstract

We report a highly bright and stable aqueous dispersion of CuInS_2_/ZnS (CIS/ZnS) nanocrystals (NCs) using surfactant-assisted microemulsion and cold treatment. CIS/ZnS NCs were facilely synthesized via a stepwise, consecutive hybrid flow reactor approach. To stabilize the optical properties of hydrophobic CIS/ZnS NCs, cetyltrimethylammonium bromide (CTAB) was chosen as a matrix for aqueous phase transfer. As the result, a high quantum yield (QY) of 56.0% and excellent photostability were acquired in aqueous media. For removing excessive surfactants, cold treatment (4°C) of the CTAB-water solution was adopted to prevent further agglomeration of CIS/ZnS NCs, which could secure high stability over 6 months (less 2% reduction in QY). The optical features and structure of the obtained CTAB stabilized CIS/ZnS (CTAB-CIS/ZnS) NCs have been characterized by UV–vis and photoluminescence (PL) spectroscopies, XRD, XPS, EDX, and TEM. The high stability and PL of water soluble CTAB-CIS/ZnS NCs suggest their potential in nanoelectronics and bioapplications.

## Background

Among the several types of inorganic nanocrystals (NCs), CuInS_2_ (CIS) NCs have been receiving enormous interests due to their non-toxic behavior and eco-friendly properties of in comparison with CdSe, CdS, PbSe, and PbS NCs containing Cd and Pb atomic pollutants. While the Restriction of Hazardous Substances Directive forbids the use of compound semiconductors containing Cd and Pb atoms in devices, the usage of CIS nanomaterials are permitted [1]. Therefore, this material might be a promising candidate for optical imaging, which offers the opportunity to develop semiconductor NCs without the toxicity limitations encountered by II-VI NCs, especially at low concentrations. CIS is an I-III-VI_2_ semiconductor with a direct band gap of 1.5 eV, corresponding to an 827 nm emission wavelength. In addition, this CIS NCs provides multicolor photoluminescence (PL) emission ranging from the visible to the NIR wavelengths (600 to 900 nm) [2].

Versatile water-soluble semiconductor NCs have been widely used for bioprobes in life science due to their benefits [3-5] of PL over conventional organic dyes: tunable PL, high photostability, long luminescence lifetime, and attractive spectrum with narrow emission and broad excitation. Although many important results have been achieved in the field of bioapplications, basic studies still need to be carried out further, such as effects of the surrounding on the PL of water-soluble quantum dot NCs. In recent years, the previous work presented the effects of pH [6], ligands [7], and excitation wavelength [8] on the optical properties of water-soluble NCs for bioprobes [9]. Therefore, a phase transfer from oil to water is imperative to ensure the water solubility of the CIS NCs before further biological applications. Moreover, the fluorescent labels for bioimaging should possess several qualities including good photochemical stability, excellent water solubility, and controlled particle size, which should be small enough to avoid possible accumulation in the body and to enhance the transportation ability in cells [4,10].

Over the past decade, several techniques such as silica shell capping [11], ligand exchange [12], and amphiphilic polymer coating [13] have been promoted to make hydrophobic nanoparticles water soluble [14]. The ligand exchange approach is easy to perform, but the resulting water-soluble quantum dot NCs are only stable for a short period, and its quantum yield (QY) decreases significantly [4] because the original hydrophobic surface ligands are replaced by hydrophilic ligands such as thioglycolic acid (TGA). Another method is to coat a hydrophilic shell such as a silica capping [12] on the surface of semiconductor NCs. This method also results in low quantum yield. In addition, the sensitivity of the silica shell to pH may cause precipitation and gel formation, and the silica shell is relatively thick and as irregular as the polymer trapping layers [15,16].

It was noted that there were also some reports involving the effect of surfactants on the PL of semiconductor NCs. Hamity and co-workers observed the PL quenching of CdS NCs by several different surfactants, e.g., cetyltrimethylammonium chloride (CTAC), Triton X-100, and sodium dodecyl sulfate (SDS), based on different quenching processes [17]. Particularly, several studies have reported the quenching effect of CTAB on the PL of CdTe NCs and observed particle aggregation in the presence of CTAB [18,19]. Using micelles or amphiphilic compounds as coating materials, the best literature results report a reduction of the PL QY for CIS/ZnS NCs in the range of 30% to 50% after transfer to water [2,20,21]. However, our study reveals that water-soluble CIS/ZnS NCs coated with CTAB possess excellent physiochemical properties conserving PL and high colloidal stability.

## Methods

### Synthesis of CIS/ZnS NCs

All chemicals were purchased from commercial sources. CIS/ZnS NCs were synthesized according to the modified procedure described in the literature [22]. In a typical synthetic procedure of CIS NCs with [Cu]/[In] molar ratio of 0.5, CuI (0.0476 g, 0.25 mmol) and In(OAc)_3_ (0.1460 g, 0.5 mmol) were mixed with n-dodecanethiol (DDT, 5 mL) in a 100-mL two-necked flask, which was followed by the addition of 1-octadecene (ODE, 4 mL). The reaction mixture was degassed under vacuum for 10 min at 150°C. Next, the solution was heated to 210°C for 1 min under nitrogen flow until a deep red colloidal solution was formed. Afterward, the reaction solution was cooled to room temperature. To make the Zn and S precursor mixture in flask, 0.734 g of zinc acetate (4 mmol) was dissolved in 4 mL of ODE with 2 mL of oleic acid (OA), then mixed with 1 mL of DDT. The resulting mixture was stirred at 100°C for several minutes under vacuum. After cooling down to room temperature, pump (1 mL/min) carried the solution (CIS cores plus Zn and S precursors) to furnace (320°C), where ZnS shells were grown on the CIS cores. Finally, the resultant CIS/ZnS core/shell NCs solution was collected at the exit of furnace. The solutions were precipitated with an excess of methanol and 1-butanol (1:1). The flocculent precipitate was centrifuged at 4,000 rpm for 2 min and the supernatant decanted. This process was repeated a minimum of three times, and the precipitation was then dried to powder for the next experiment and characterization.

### Phase transfer of CIS/ZnS NCs to aqueous phase

**Figure 1:**
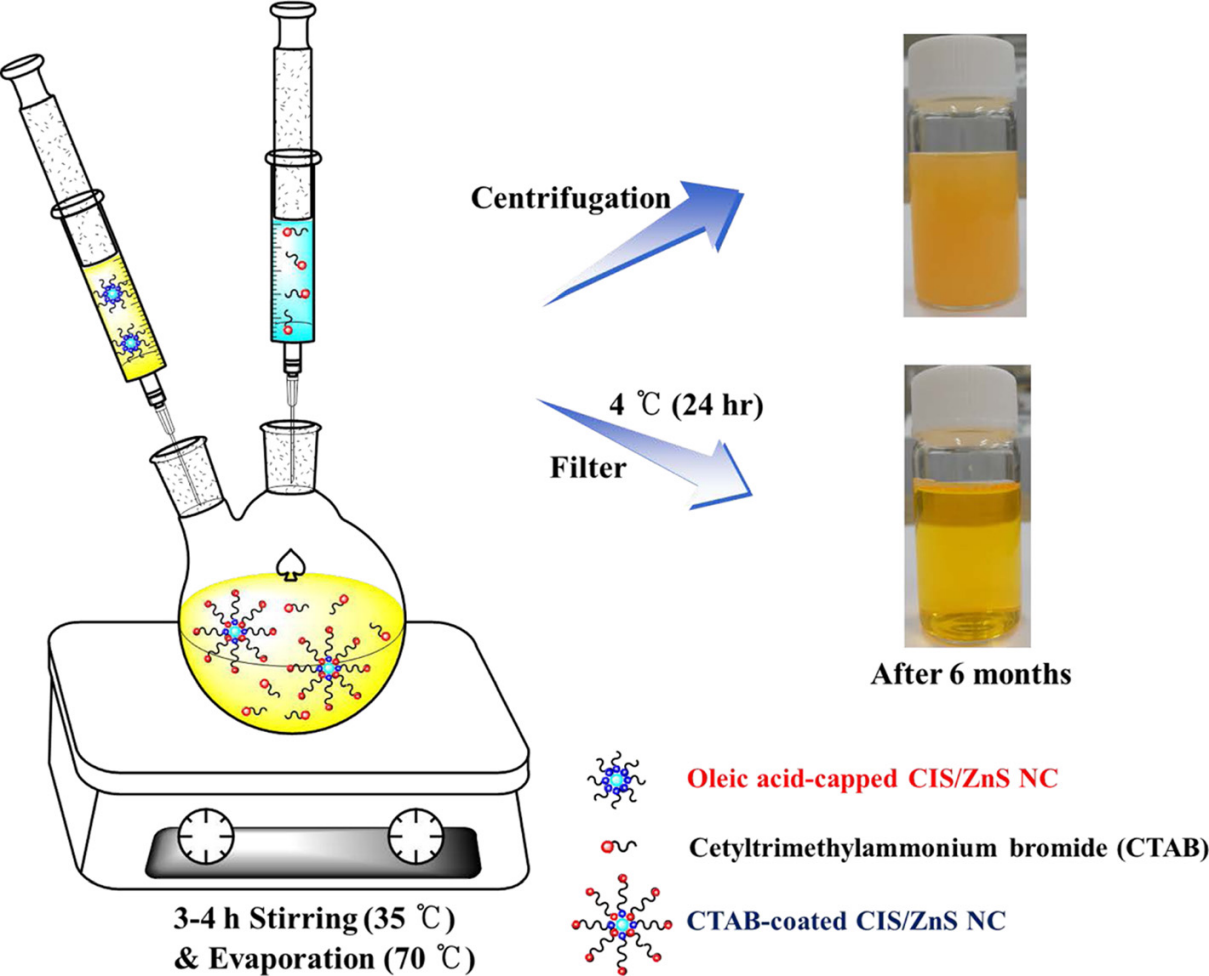
**Synthesis scheme of CTAB-CIS/ZnS NCs.**

The CIS/ZnS NCs (0.12 g in 4 mL of chloroform) were added to aqueous CTAB solution (0.8 g in 40 mL of H_2_O). The resulting solution was stirred vigorously at 35°C for 3 to 4 h. The formation of an oil-in-water microemulsion resulted in an opaque solution. The solution was then transferred to a heating mantle at 70°C for 10 min to evaporate the chloroform as well as to induce the interaction between the hydrophobic chains of the two surfactants. After cooling down to room temperature, it was put in the fridge (4°C) for 24 h. The NC solution was then further filtered with a 0.25 μm syringe filter to remove excess CTAB. The prepared water soluble CTAB-CIS/ZnS NCs were stored for subsequent experiments in sealed dark conditions. The procedure for the fabrication of the CTAB-CIS/ZnS NCs is described schematically in Figure 1.

### Preparation of CTAB-CIS/ZnS NC films by spray process

Glass substrates were placed on a hot plate and secured in place by masking tape. A commercially available spray gun (Kinki Creamy, Kinki Marketing, Georgetown, Penang, Malaysia) was placed directly over the substrate at a fixed distance of 20 cm. The spray gun was attached to a pressurized nitrogen gas cylinder with a constant pressure of 0.35 MPa. The nozzle aperture was adjusted by a screw at the back of the gun. Spraying for all samples was done at constant pressure, distance, and concentration. The spray time for each sample was 10 min. The substrate was heated to 50°C during the spray process using a hot plate to accelerate evaporation of the solvent.

### Characterization

Optical characterization of the oil soluble CIS/ZnS NCs and the water soluble CTAB-CIS/ZnS NCs was carried out using a UV–vis spectrophotometer (Optizen 2120, Mecasys Co., Ltd., Daejeon, Korea), a fluorometer (SV2100-F, K-MAC, Daejeon, Korea) and an absolute QY measurement system (C-9920-02, Hamamatsu Photonics, Hamamatsu, Japan). X-ray diffraction analysis was performed using a D/MAX Ultima III diffractometer (Rigaku, Tokyo, Japan) operated at a 40 kV voltage and 40 mA current with Cu Kα radiation. High-resolution transmission electron microscopy (TEM) was obtained using a Tecnai G2 F30 S-Twin model (FEI, Hillsboro, OR, USA) at 300 kV. The photoelectron spectra were obtained with a ESCA-2000 Multilab apparatus (VG Microtech, Sussex, UK) using a nonmonochromatic Mg Kα excitation source and a hemispherical analyzer.

## Results and discussion

**Figure 2:**
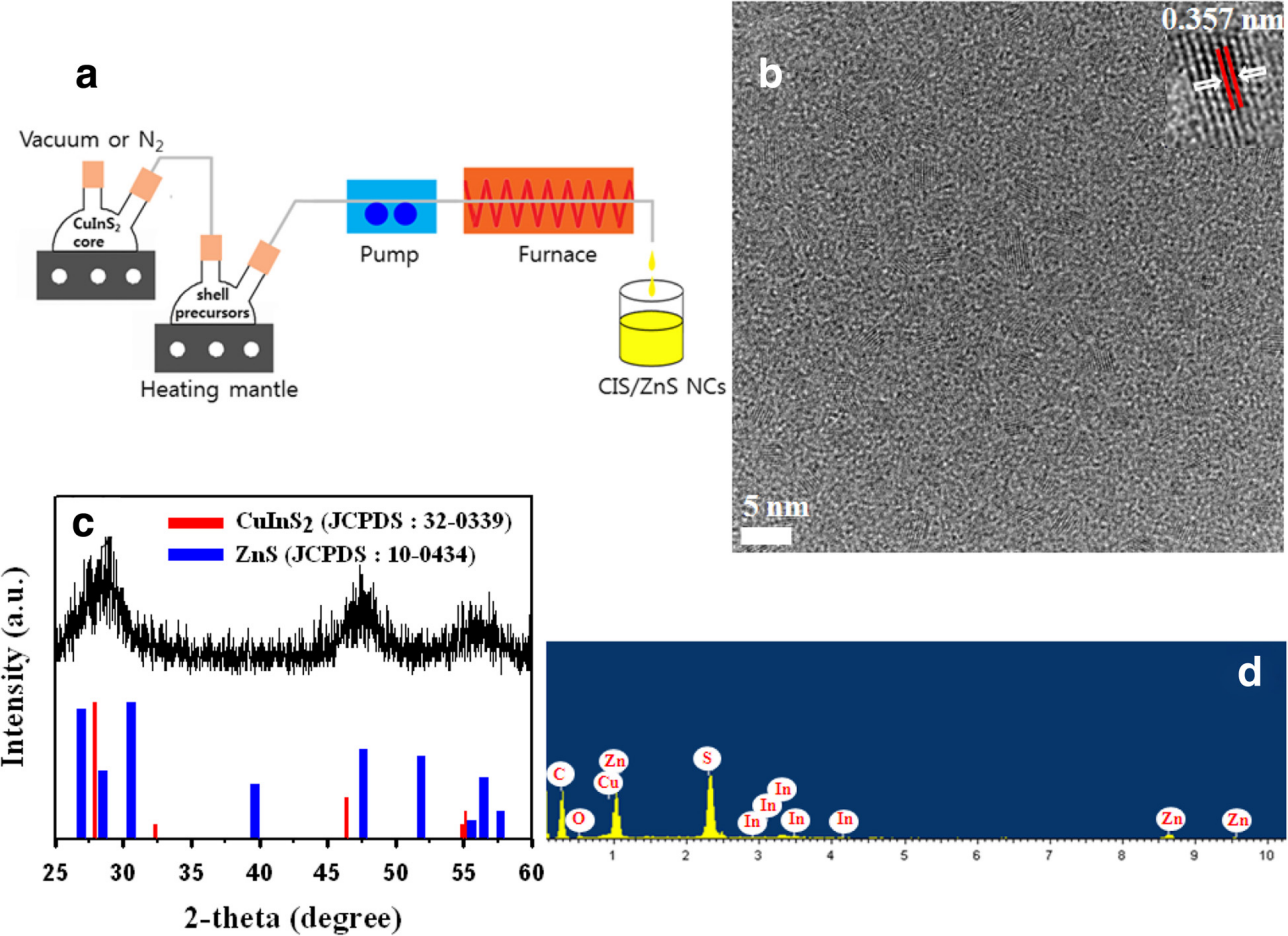
**Schematic diagram and structural analysis of CIS/ZnS NCs. (a)** Schematic diagram showing the hybrid flow reactor. **(b)** High-resolution TEM image of CIS/ZnS NCs. **(c)** X-ray diffraction pattern of CIS/ZnS NCs. **(d)** EDX spectrum of the CIS/ZnS NCs.

The scheme of our hybrid flow reactor is shown in Figure 2a. The reactor is composed of two flask mixers, one pump and one furnace. Both flow rate and temperature can be controlled. Owing to the facileness of our stepwise, consecutive hybrid flow reactor approach, CIS/ZnS NCs are also readily scalable to a larger amount. This method can produce gram quantities of material with a chemical yield in excess of 90% with minimal solvent waste. The detailed experimental procedure is provided in the ‘Methods’ section. As shown in Figure 2b, the CIS/ZnS NCs were quasispherical particles with an average diameter of about 4 to 5 nm. The existence of well-resolved lattice planes in the inset of Figure 2b demonstrates the good crystallinity of the NCs; moreover, the lattice spacing between two adjacent planes was 0.357 nm. Figure 2c shows the XRD pattern of the as-prepared CIS/ZnS core/shell NCs. The powder patterns for CIS (red color) and ZnS (blue color) are also shown for comparison in the bottom to inset. The location of the pattern is in good agreement with the Joint Committee on Powder Diffraction Standards (JCPDS) reference diagrams in the bottom inset (JCPDS No. 32–0339: CuInS_2_ and 10–0434: ZnS). EDX measurement result shown in Figure 2d indicates that the NCs were composed of copper, indium, zinc, and sulfur elements.

**Figure 3:**
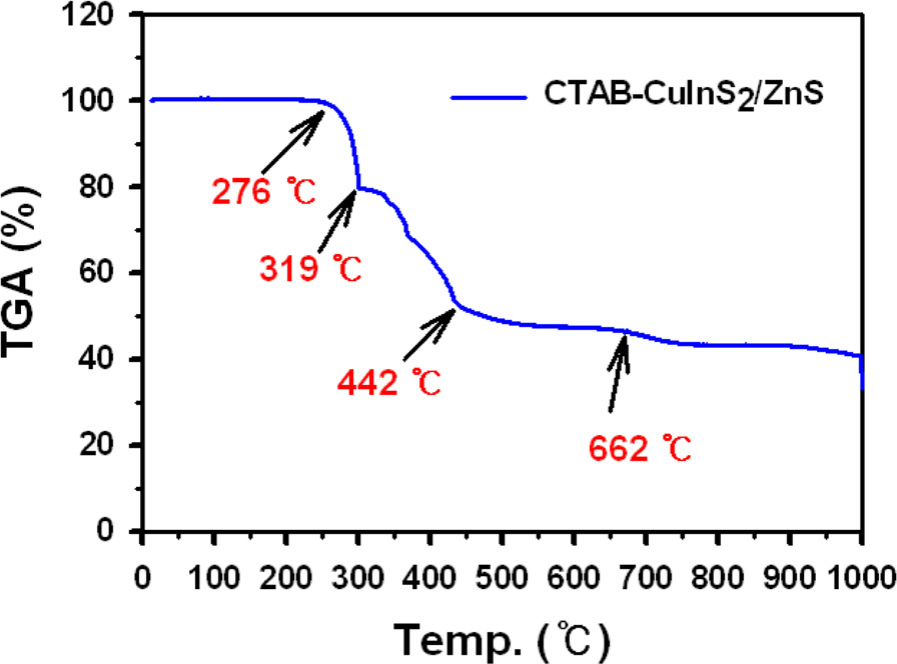
**TGA data of CTAB-CIS/ZnS NCs.**

The yield of the nanocrystals after excess CTAB removal was around 90%. The TGA data is shown Figure 3. Corresponding TGA curve exhibits three weight losses of 21%, 29%, and 10% at 276°C to 319°C, 319°C to 442°C, and 442°C to 662°C, respectively. The last two weight-loss steps in the temperature range of 319°C to 662°C, which are resulted from decomposition of CTAB, reflect two different statuses of CTAB capped on CIS/ZnS NCs, suggesting the formation of CTAB bilayer on the metal surface. For the peak at 276°C, which is concomitant by a less than 21% weight loss, it is believed to be desorption of bromine and nitrogen entrapped between CIS/ZnS NCs and CTAB bilayer. In the range from 319°C to 662°C, the first sharp step of 29% weight-loss arises within 319°C to 442°C and is considered as decomposition of CTAB outer layer. While the second step of 6% weight-loss in 442°C to 662°C is attributed to CTAB inner monolayer decomposition. The ratio of molecules in inner and outer layers is thus roughly estimated as 1:5 by comparing the amount of the weight-losses from these two steps. On the basis of this measured results, the total amount of CTAB molecules both in adsorbed bilayers and in the free state is approximately matchable to the calculated value, according to CIS/ZnS NCs’ size and CTAB head group area.

**Figure 4:**
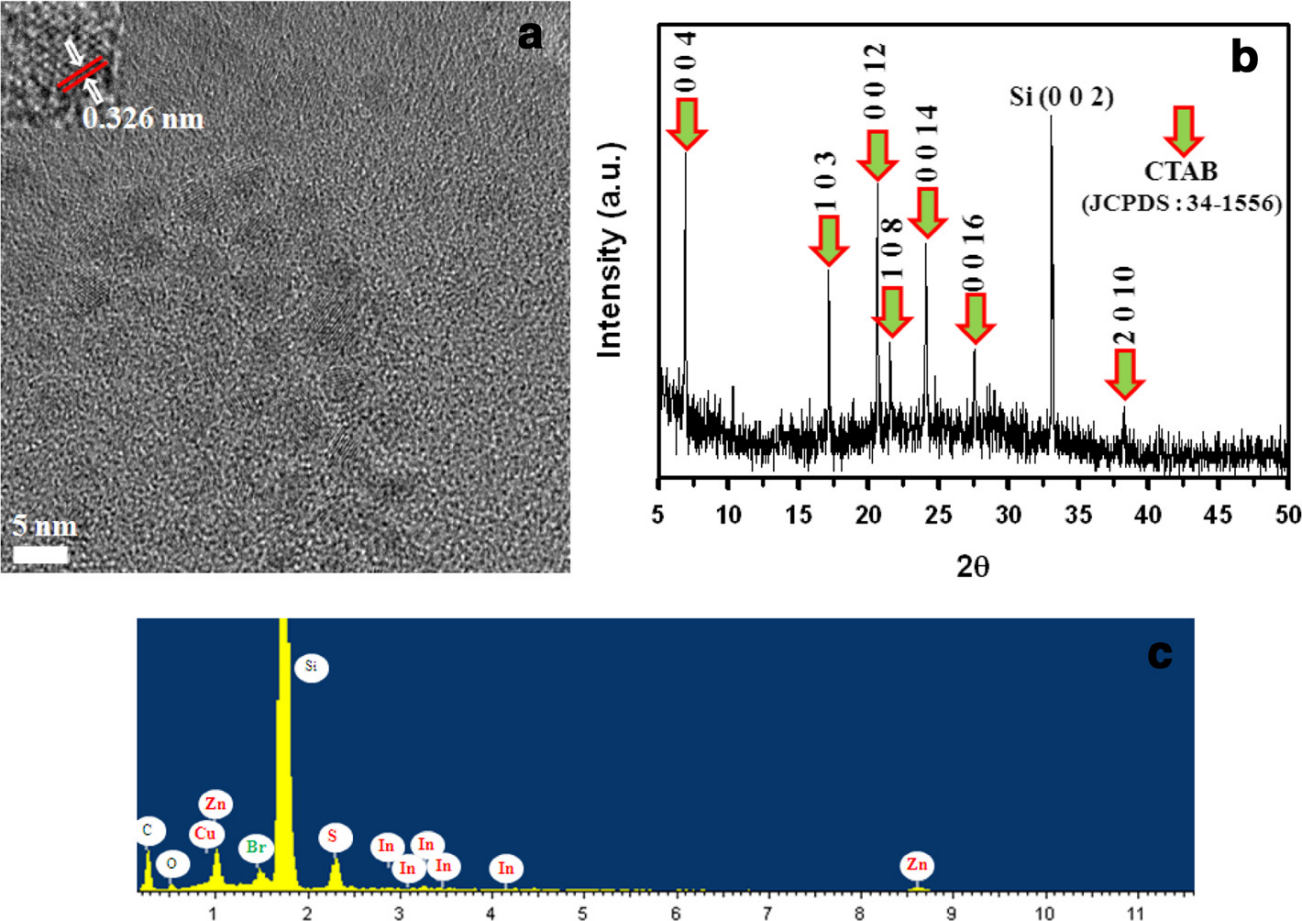
**Structural analysis of CTAB-CIS/ZnS NCs. (a)** High-resolution TEM image of CTAB-CIS/ZnS NCs. **(b)** X-ray diffraction pattern of CTAB-CIS/ZnS NCs. **(c)** EDX spectrum of the CTAB-CIS/ZnS NCs.

The nanostructure of the CTAB-CIS/ZnS core-shell NCs was observed from conventional HRTEM as shown in Figure 4a. From the TEM image, the size of core-shell can be estimated in the range of 4 to 5 nm. The average crystallite size calculated from the water soluble CTAB-CIS/ZnS NCs was found to be consistent with those obtained from the TEM result of the oil soluble CIS/ZnS NCs (Figure 2b). The XRD pattern of the synthesized CTAB-CIS/ZnS NCs is shown in Figure 4b. The XRD peaks at 6.94°, 17.18°, 20.62°, 21.52°, 24.12°, 27.58°, and 38.3° correspond to the crystal planes (0 0 4), (1 0 3), (0 0 12), (1 0 8) (0 0 14), (0 0 16), and (2 0 10) of the monoclinic CTAB phase (JCPDS card no. 34–1556), respectively. Besides the characteristic peaks of the CTAB as shown Figure 4b, a strong and sharp peak around 2θ = 33.18° derived from the silicon wafer can be observed, suggesting the successfully coating of CTAB on the surface of CIS/ZnS nanoparticles. The energy-dispersive X-ray spectroscopy (EDX) analysis was performed to confirm the chemical stoichiometry and the successful coating of CTAB in the CIS/ZnS core-shell NCs. From Figure 4c, the Br element is clearly indicated together with Cu, In, Zn, and S elements. It should be noted that the origin of strong Si peak that appeared in the EDX spectrum is from the silicon wafer. The C and O elements also came from the capping agents (OA and CTAB). The EDX analysis of NCs provides an additional evidence of the synthesis water soluble CTAB-CIS/ZnS NCs.

**Figure 5:**
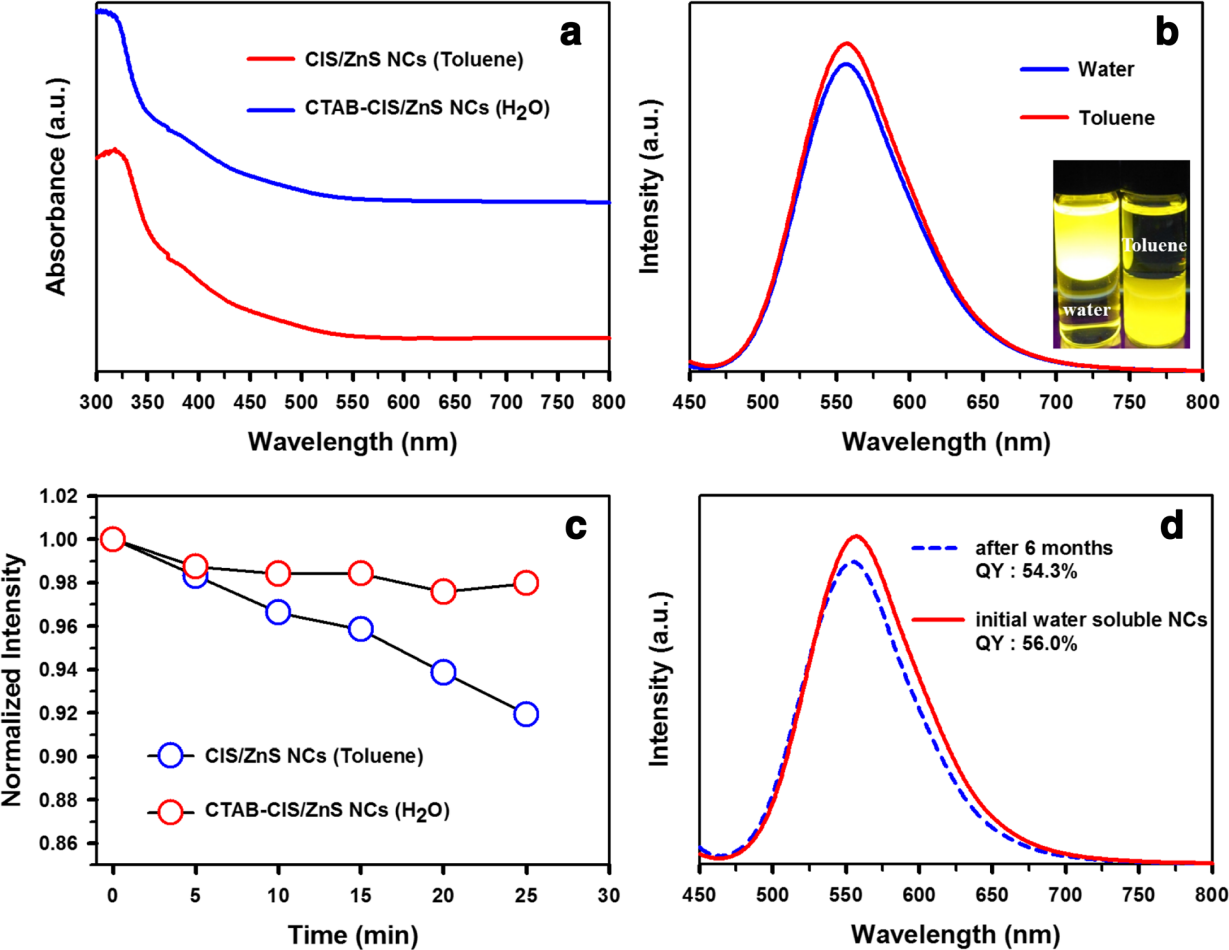
**Optical properties of CTAB-CIS/ZnS NCs. (a)** UV–vis absorption spectra and **(b)** PL spectra of CIS/ZnS NCs before (toluene dispersions) and after (aqueous dispersions) phase transfer using CTAB. Photograph of samples dispersed in different media under UV light is shown in the inset in the Figure 3b. **(c)** The evolution of PL intensity and **(d)** emission measurement after 6 months in the dark of CTAB-CIS/ZnS NCs.

The optical properties of the NCs before and after phase transfer were also investigated (Figure 5a). It was shown that the absorption spectra of oil soluble CIS/ZnS NCs and water soluble CTAB-CIS/ZnS NCs were similar, both exhibiting the position of the first absorption peak and the shape of absorption spectra. The PL spectrum of CTAB-CIS/ZnS NCs was nearly the same as that of the oil-soluble NCs with 556.7 nm wavelength (Figure 5b), exhibiting a relatively high QY of 56.0% (compared with a QY of 61.4% in the organic phase), which clarifies that the CTAB coating layer maintained the emission properties of NCs regarding the PL spectrum and QY. The highly preserved QY and spectral features (without an obvious PL red shift from hydrophobic NCs as previously observed [23,24]) probably benefited from lipophilic CTAB encapsulation (hydrophobic interaction) on CIS/ZnS NCs without disturbing the originally capping ligands. This favored the surface passivation of CIS/ZnS NCs, which is critical for their optical emission properties [14]. The colors of the solutions before and after phase transfer were nearly identical, and both a nonpolar solvent and aqueous solution displayed bright yellow luminescence under UV irradiation at 365 nm (inset of Figure 5b). With the continual exposure to UV light (Figure 5c), a bare CIS/ZnS NCs exhibited a steadily reduced PL intensity. On the other hand, the PL intensity of CTAB-CIS/ZnS NCs was gradually decreased during the initial operational times and then rather enhanced. To account for the different PL stability behaviors observed, the role of CTAB matrix was explained in conjunction with photoinduced oxidation of CIS/ZnS NCs. Notably, CTAB-embedded NCs exhibited good colloidal stability and photostability in water. No precipitations or obvious decrease in the luminescent intensity was observed after the aqueous solution was kept at room temperature in the dark for 6 months (Figure 5d). Besides yield and stability, the QY was another concern as the decrease of QY in phase transfer was commonly reported for various NCs [25-28]. However, in this study, the QYs of CIS/ZnS NCs and CTAB-CIS/ZnS NCs were determined to be 61.4% and 56.0%, respectively, indicating that the surface passivation with CTAB had little effect on the luminescent intensity or QY of the NCs. The luminescence QY of the water soluble CTAB-CIS/ZnS NCs was decreased to 1.7% after 6 months of reaction time. These results suggest that the long-term stability of the PL might be attributed to appropriate coating of the CTAB chains, leading to a more efficient insulating barrier on the surface of the CIS/ZnS NCs [29].

**Figure 6:**
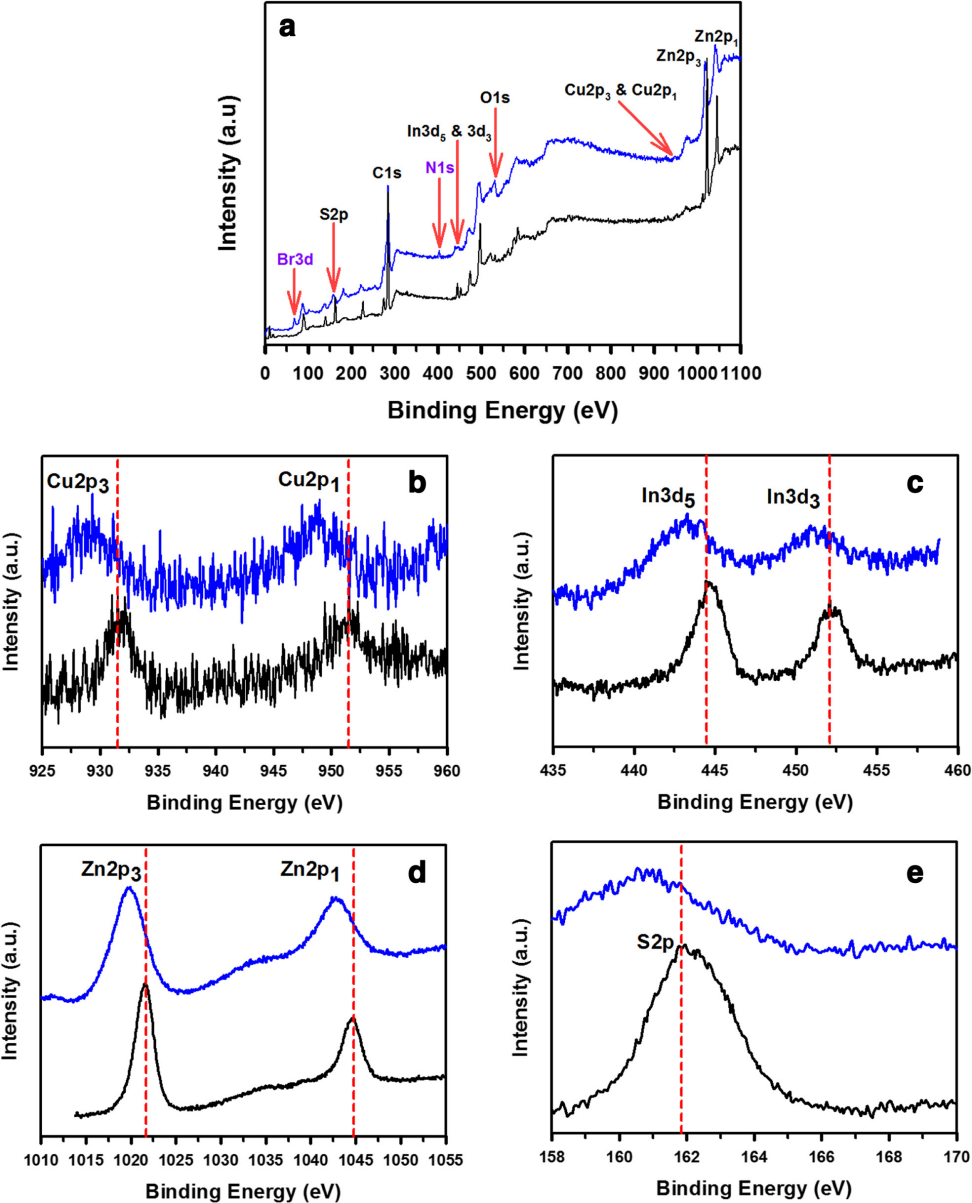
**XPS spectra of the original (black lines) and CTAB-CIS/ZnS NCs (blue lines). (a)** Survey spectra, **(b)** Cu 2p, **(c)** In 3d, **(d)** Zn 2p, and **(e)** S 2p.

In order to make clear the causes of the enhanced photostability for the CTAB-CIS/ZnS NCs, we examined the surface change of the nature of the CIS/ZnS NCs before and after CTAB treatment using X-ray photoelectron spectroscopy (XPS) and the results were shown in Figure 6. According to the survey spectra (Figure 6a), the elements Cu, In, S, Zn, Br, N, O, and C were all detected, proved the existence of these elements in the sample. The survey spectrum of the CTAB-CIS/ZnS NCs (blue line) showed the remained characteristic peaks of Cu 2p, In 3d, S 2p, and Zn 2p, implying that the coating of CTAB gave no significant influence on the crystal particles. The C, O, Br, and N are derived from the capping agents (OA and CTAB) on the surface. Moreover, the peak positions and shapes of Cu 2p (929.3 and 949.0 eV, respectively, for Cu 2p3/2 and 2p1/2), In 3d (443.3 and 450.9 eV, respectively, for In 3d5/2 and 3d3/2), Zn 2p (1019.9 and 1042.8 eV, respectively, for Zn 2p3/2 and 2p1/2), and S 2p (160.6 eV) were observed. The spectra of the Cu 2p, In 3d, Zn 2p, and S 2p level were significantly broadened and shifted by 1.2 to 2.5 eV to lower binding energy after the CTAB coating. These results provided evidence that in all probability a new CTAB-CIS/ZnS NCs with a lower binding energy was formed on the surface of CIS/ZnS NCs after the treatment of CTAB, in which the CTAB layers passivate the surface defects very effectively [30,31], and the resulting NC shows an enhanced photostability.

**Figure 7:**
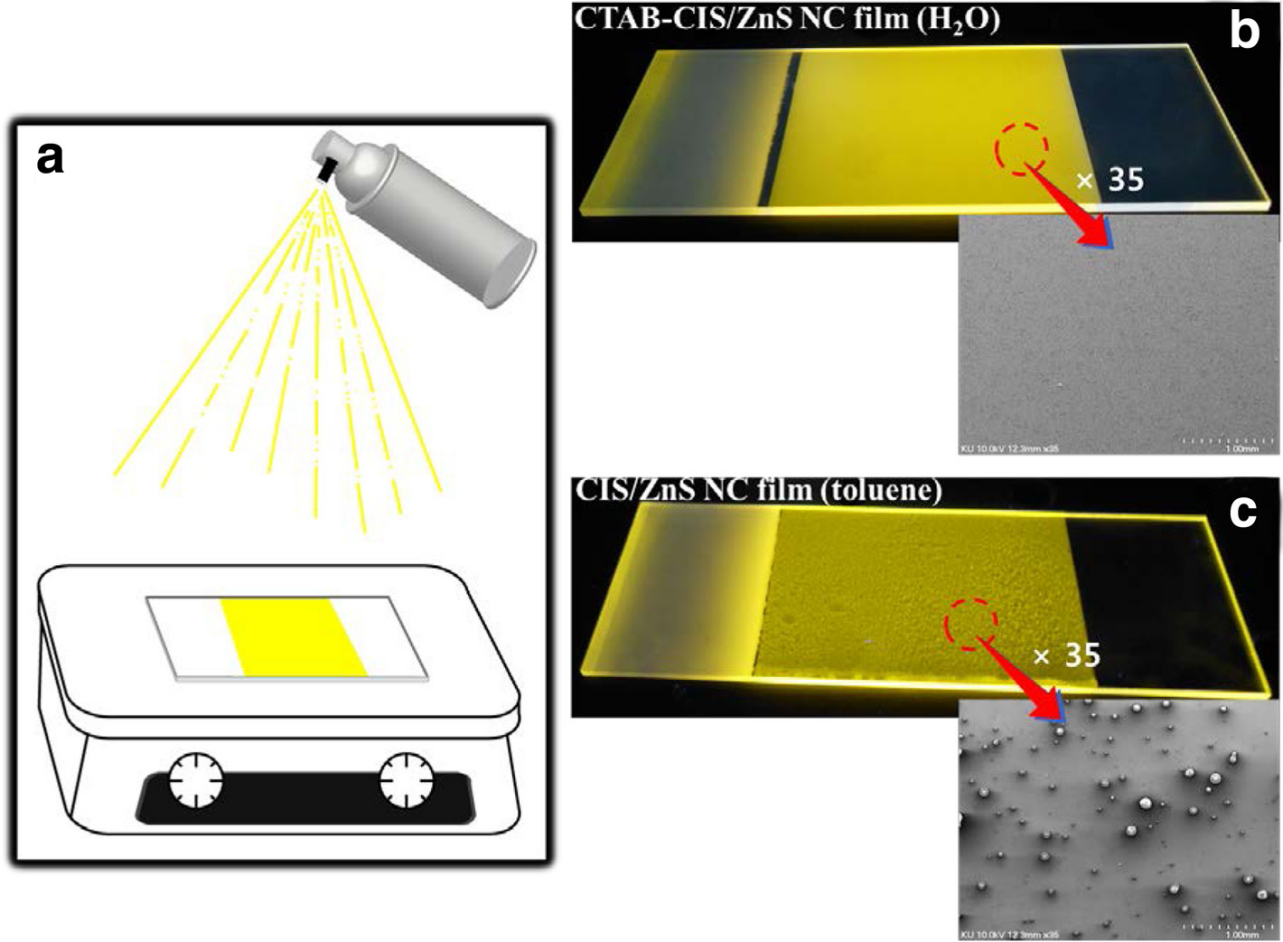
**Spray process and photos of samples under irradiation of 254-nm light. (a)** Scheme of spray process and photos of films coated on glass substrates; **(b)** CTAB-CIS/ZnS NC film (water) and **(c)** CIS/ZnS NC film (toluene).

The NC films have been prepared by a spray coating method on glass surface. Figure 7a shows the spray process. Heat-treatment of the substrates plays an important role to obtain homogeneous films. The substrates were heated to 50°C, followed by coating NCs on them, where the solvent was steadily evaporated to form a film on the substrate surface. Such low temperature did not affect the PL efficiency of the NCs during the spray process [32]. Figure 7b,c shows photos of samples under irradiation of 254 nm light. Figure 7b shows the photo of film with water soluble CTAB-CIS/ZnS NCs. The NCs were uniformly dispersed in the film without aggregation. Homogeneous film was coated on the glass substrate. The bright PL was observed. However, the fluorescence of the oil soluble CIS/ZnS NC film looks like a heterogeneous surface (Figure 7c), which indicates that the NCs dissolve in the toluene if they are not heated at a high temperature during spray process [33]. As a result, the CTAB-CIS/ZnS NC film can be applied to biosensing assemblies, biomedical devices, and light-emitting devices.

## Conclusions

In summary, we have demonstrated that CIS/ZnS NCs synthesized on a large scale using a hybrid flow reactor in a simple, one-step process can be effectively transferred into aqueous solution by adding CTAB. UV–vis absorption spectra and fluorescence spectra showed that the obtained CTAB-CIS/ZnS NCs present good optical properties. The QY of the cluster ranged up to 56.0% in water using core-shell CIS/ZnS NCs with QY of 61.4% in nonpolar solvent. The high QY of the CIS/ZnS NCs was well maintained after the phase transfer. In addition, the photostability of CTAB-CIS/ZnS NCs was investigated at ambient condition under UV irradiation. It was found that the yellow PL exhibited by the composite was very stable: the intensity, position, and shape show very little change even after being aged over 6 months. Cold treatment of the CTAB aqueous micellar solution plays an important role to obtain stable state. The CTAB-CIS/ZnS NCs have desirable advantages such as high PL QY, good colloidal stability, and photostability, and thus may have great potential for versatile applications in biological labels and light-emitting diodes.
